# Nanotension Relief Agent Enhances Tissue Penetration by Reducing Solid Stress in Pancreatic Ductal Adenocarcinoma via Rho/ROCK Pathway Inhibition

**DOI:** 10.34133/bmr.0173

**Published:** 2025-04-09

**Authors:** Feiran Yu, Gaorui Zhang, Jintang Sun, Yuxuan Zhao, Yafei Qi, Xiaoyu Han, Chen Ai, Weikai Sun, Jiazhi Duan, Dexin Yu

**Affiliations:** ^1^ Department of Radiology, Qilu Hospital of Shandong University, Jinan, Shandong 250012, China.; ^2^ Translational Medicine Research Center in Nano Molecular and Functional Imaging of Shandong University, Jinan 250012, China.; ^3^ Research Center for Basic Medical Sciences, Qilu Hospital of Shandong University, Jinan, Shandong 250012, China.; ^4^ Institute for Advanced Interdisciplinary Research, University of Jinan, Jinan 250022, China.

## Abstract

The formidable contractile tension exerted by cancer-associated fibroblasts (CAFs) in pancreatic ductal adenocarcinoma (PDAC) tissue is crucial for maintaining high tissue solid stress (TSS), which impedes the delivery and penetration of chemotherapeutic drugs. To address this obstacle, we constructed a pH-responsive nanotension relief agent (FS@MMS), in which fasudil (FS) was ingeniously conjugated to mesoporous silica encapsulated with magnetic iron oxide (MMS). The nanotension relief agent was demonstrated to inhibit the synthesis of phosphorylated myosin light chain by blocking the Rho/Rho-associated serine/threonine kinase (ROCK) pathway, triggering the swift transformation of high-tension CAFs into low-tension CAFs in PDAC tissue, which relieves TSS and enhances drug penetration in Panc02/NIH-3T3 multicellular tumor spheroids. When the nanotension relief agent was further loaded with the chemotherapeutic drug gemcitabine (GEM), as FS@MMS-GEM, the enhanced permeation of GEM progressively killed tumor cells and amplified their TSS-relief properties, thereby maximizing the anticancer efficacy of chemotherapeutic agents in Panc02/NIH-3T3 coplanted model mice. The magnetic resonance imaging results revealed that the synergistic effect substantially improved drug delivery and penetration efficiency. The developed approach holds great potential for improving chemotherapy efficacy in PDAC and provides a novel therapeutic approach for the treatment of related stroma-rich tumors.

## Introduction

Pancreatic ductal adenocarcinoma (PDAC) is one of the most fatal malignant cancers worldwide; the 5-year survival rate is only approximately 10%, despite advancements in chemotherapy [1,2]. The degree of tissue solid stress (TSS) within PDAC tissues is much greater than that in normal pancreatic tissue, posing an important barrier to the effectiveness of chemotherapeutic drugs [3]. This stress arises from the mechanical forces caused by rapid proliferation of tumor cells and stroma remodeling and hardening [4,5]. A high TSS can hinder the transport and penetration of traditional chemotherapeutic drugs within PDAC tissue [6]. Consequently, alleviating the TSS and reshaping the tumor mechanical microenvironment are indispensable prerequisites for improving treatment efficiency in PDAC tissue.

Cancer-associated fibroblast (CAF)-mediated stroma remodeling is essential for the increase in solid stress levels in patients with PDAC. Compared with normal fibroblasts, CAFs exhibit a heightened capacity to synthesize substantial quantities of extracellular matrix (ECM) proteins and interstitial enzymes. This robust production facilitates profound interstitial remodeling processes and the development of sclerosis. Furthermore, CAFs are capable of manifesting an abundant array of stress fibers, which harness their contractile ability to exert powerful tensile forces on the fibrous stroma. This mechanical pulling action intensifies interstitial densification. Notably, the ensuing rigid interstitial fibrosis, in turn, acts as a pivotal stimulus for CAF activation, thereby establishing a perpetuating positive feedback loop to relentlessly increase the TSS [7–10]. Based on this characterization, breaking the vicious cycle of interstitial remodeling and hardening appears to be a potential strategy to relieve TSS. Nanoparticle albumin-bound paclitaxel combined with gemcitabine (GEM) can nonspecifically kill CAFs to reduce fiber and TSS in PDAC tissues indirectly, but this regimen has shown limited success in extending patient survival by only a few months [11–13]. Nanomedicines aimed at stroma depletion have demonstrated poor long-term efficacy due to off-target effects and increased tumor aggressiveness [14,15]. Therefore, the optimal strategy should break the vicious cycle of increased TSS and potentiate the deep penetration of chemotherapy drugs while maintaining PDAC matrix homeostasis.

Previous studies have shown that transient Rho-associated serine/threonine kinase (ROCK) activity can reverse the hypercontractile phenotype of CAFs in the long term [16]. High-tension CAFs are responsible for generating TSS in PDAC tissue, and the Rho/ROCK pathway is hyperactivated in CAFs, enabling the promotion of cell contractile force production through the regulation of myosin light chain (MLC) phosphorylation [17,18]. Thus, inhibiting ROCK protein phosphorylation can inhibit actomyosin-mediated contraction in CAFs, achieve interstitial loosening, and reduce TSS in a short time. At present, fasudil is the first clinically approved selective ROCK inhibitor. However, the conventional intravenous administration of fasudil has several limitations, including low accumulation in PDAC tissue, a short blood clearance half-life, and a narrow treatment window [19,20]. These limitations reduce drug accumulation in PDAC tissues and increase side effects, ultimately leading to unsatisfactory treatment outcomes.

**Fig. 1.**
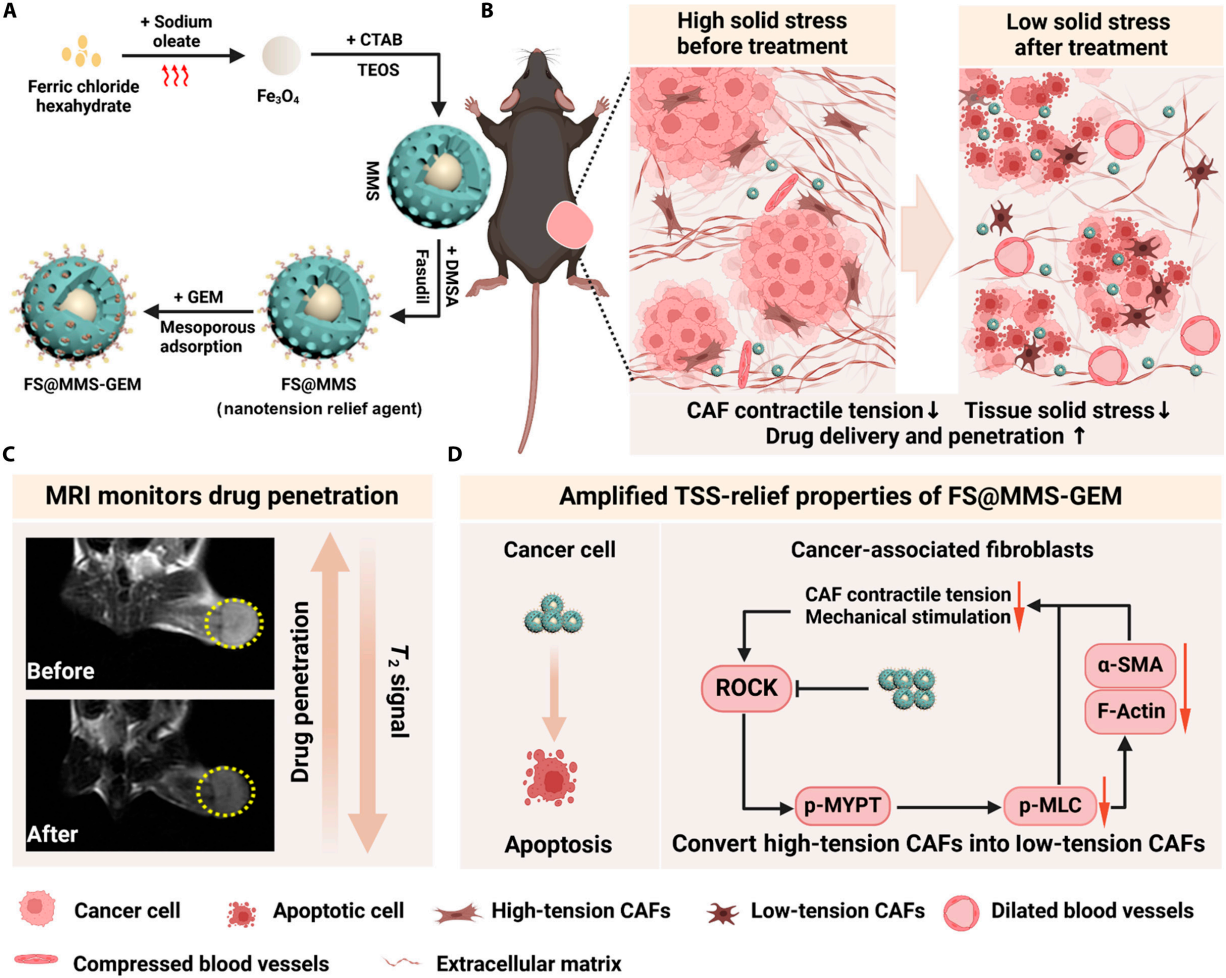
Tissue solid stress (TSS) relief scheme for pancreatic ductal adenocarcinoma (PDAC). (A) Schematic diagram illustrating the generation of FS@MMS-GEM. (B) FS@MMS-GEM treatment reduced the TSS in PDAC tissue and enhanced drug penetration, which increased the anticancer effect of chemotherapeutic agents. (C) Magnetic resonance imaging (MRI) was used to assess the drug penetration efficiency. (D) Fasudil inhibited phosphorylated myosin light chain (p-MLC) synthesis by blocking the Rho/Rho-associated serine/threonine kinase (ROCK) pathway, resulting in the rapid conversion of high-tension cancer-associated fibroblasts (CAFs) to low-tension CAFs, which reduced TSS and increased drug penetration efficiency. The enhanced permeation of gemcitabine (GEM) progressively killed tumor cells and amplified the TSS-relief properties of FS@MMS-GEM. Created with BioRender.com. CTAB, cetyltrimethylammonium bromide; TEOS, tetraethoxysilane; MMS, mesoporous silica encapsulated with magnetic iron oxide; α-SMA, alpha smooth muscle actin; p-MLC, phosphorylated myosin light chain; DMSA, dimercaptosuccinic acid; p-MYPT, phospho-myosin phosphatase.

In this study, we developed a novel strategy aimed at reducing the contractile tension of CAFs on interstitial fibers to alleviate TSS in patients with PDAC. Mesoporous silica is a widely studied biological carrier that has high biosafety, high loading efficiency, and easy modification characteristics [21,22]. Fasudil was linked to mesoporous silica via dimercaptosuccinic acid (FS@MS). As shown in Fig. 1, to monitor drug penetration in real time, we further integrated magnetic iron oxide into this formulation, yielding FS@MMS, which we term the “nanotension relief agent”. After the nanotension relief agent accumulates in PDAC tissue, fasudil is liberated in the acidic microenvironment, which triggers the inhibition of the Rho/ROCK signaling pathway, ultimately halting actomyosin contraction within CAFs. The transition from high-tension to low-tension CAFs diminishes mechanical stimulation, thereby preventing the excessive synthesis of stress fibers and the accumulation of ECM proteins. In this way, nanotension relief agents break the feedback loop that increases the TSS. When GEM was efficiently loaded into the mesopores of FS@MMS (FS@MMS-GEM), the nanotension relief agent facilitated GEM penetration and accumulation within PDAC tissues. The subsequent release of GEM exerts its cytotoxic effects, synergizing with the tension-relieving properties of FS@MMS to amplify the TSS-relief characteristics of FS@MMS-GEM. This synergistic action maximizes the antitumor effect. In brief, our optimized nanostrategy inhibits intracellular actomyosin contraction in CAFs, which relieves TSS and improves therapeutic efficacy via multiple mechanisms. This innovative approach enhances drug penetration and chemotherapeutic efficacy by relieving TSS without resorting to the direct elimination of tumor interstitial tissue. Consequently, it offers a hopeful avenue for the treatment of various stromal-rich tumors, presenting a feasible and potentially transformative therapeutic strategy.

## Materials and Methods

### Materials

Hexane, sodium oleate, cetyltrimethylammonium bromide (CTAB), and tetraethoxysilane were purchased from Sinopharm Chemical Reagent Co., Ltd. (Shanghai, China). Transforming growth factor-β (TGF-β) was purchased from PeproTech. p-MLC2 (Ser19) and phospho-myosin phosphatase 1 (p-MYPT1) (Thr696) antibodies were purchased from Cell Signaling Technology (USA). Anti-alpha smooth muscle actin (α-SMA) antibody (ab7817) and anti-collagen I antibody (ab270993) were purchased from Abcam (USA). Cell Counting Kit-8 (CCK-8) was obtained from Dojindo Laboratories (Japan). The chemiluminescence horseradish peroxidase substrate was purchased from Millipore (USA).

### Cell lines and animals

Murine pancreatic adenocarcinoma (Panc02) and embryonic fibroblasts (NIH-3T3) were obtained from the Type Culture Collection of the Chinese Academy of Sciences (Shanghai, China). All cells were cultured in Dulbecco’s modified Eagle medium supplemented with 10% fetal bovine serum, 100 U/ml penicillin, and 100 μg/ml streptomycin at 37 °C in a 5% CO_2_ constant-temperature incubator.

Female C57BL/6 mice (4 to 6 weeks old) were obtained from Jinan Pengyue Experimental Animal Co., Ltd. The animal experiments were conducted following the guidelines for the care and use of animals established by the National Institutes of Health (USA) and were approved by the Animal Experiment Committee of Qilu Hospital, Shandong University. To establish the Panc02/NIH-3T3 tumor-bearing mouse model, female C57BL/6 mice were subcutaneously injected with 4.0 × 10^6^ Panc02 cells and 2.0 × 10^6^ NIH-3T3 cells in the right thigh. The mice were fed until the tumor reached the appropriate volume for the experiment. The tumor volume was calculated as *V* = *a*^2^ × *b*/2, where “*a*” represents the minimum diameter of the tumor (mm) and “*b*” represents the maximum diameter of the tumor (mm).

### Synthesis of the nanotension relief agent and FS@MMS-GEM nanoparticles

The synthesis method of the Fe_3_O_4_ nanoparticles was adopted from our previous work and is briefly described as follows: First, 60 ml of deionized water, 80 ml of ethanol, and 140 ml of hexane were sufficiently mixed. Next, ferric chloride hexahydrate (10.8 g) and sodium oleate (36.5 g) were added to the mixture [23]. The mixture was heated to 70 °C for 4 h. The upper layer of the mixture was collected and washed with distilled water 3 times. The iron oleate complex in the mixture was collected by evaporation and subsequently added to 5.7 g of oleic acid and 200 g of 1-octadecene. The mixture was heated to 320 °C for 30 min and then cooled to room temperature. The Fe_3_O_4_ nanoparticles were collected by adding a large amount of alcohol to the reaction mixture and performing further centrifugation.

The mesoporous silica encapsulated with magnetic iron oxide (MMS) particles were synthesized as follows: First, 20 mg of the obtained Fe_3_O_4_ nanoparticles was dissolved in a mixture of 5 ml of chloroform, 0.5 g of CTAB, and 40 ml of water. Fe_3_O_4_–CTAB was obtained by adequate sonication followed by chloroform evaporation. Then, 500 μl of aqueous NaOH (2 M) and 10 mg of the obtained Fe_3_O_4_–CTAB were added to 50 ml of deionized water and heated to 70 °C, after which 0.2 ml of 3-aminopropyltrimethoxysilane and 0.5 ml of tetraethoxysilane were added to the mixture. The mixture was incubated at this temperature for 30 min. The MMS nanoparticles were collected by centrifugation and washed several times with alcohol. To synthesize FS@MMS, fasudil (0.25 mg/ml) and dimercaptosuccinic acid (0.05 mg) were added to the MMS, and we termed this product the “nanotension relief agent”. Afterward, GEM (0.5 mg/ml) was added to the FS@MMS nanoparticles to synthesize FS@MMS-GEM. The reaction product was collected with centrifugation.

### Characterization of the Fe_3_O_4_ nanoparticles and nanotension relief agent

The morphology and size of the synthesized nanocarriers were characterized via transmission electron microscopy (TEM). The structures of the synthesized materials were characterized by x-ray diffraction. The surface chemical compounds of the synthesized materials were characterized by Fourier transform infrared spectroscopy (FTIR). The saturation magnetization of the synthesized nanoparticles was measured using a vibrating-sample magnetometer.

### Drug loading and drug release

A dispersion of 1 mg/ml FS@MMS-GEM was prepared in ultrapure water and centrifuged (14,000 r/min, 30 min). The supernatant was diluted to an appropriate concentration, and the contents of free GEM and fasudil were measured by ultraviolet–visible (UV–Vis) spectrometry at 270 and 321 nm. The encapsulation rate and drug loading capacity were calculated by the following formulas:

$$\begin{array}{c} \text{Encapsulation rate}\;\left(\%\right)=\\ \frac{\text{sample quantity}\;-\;\text{free drug quantity}}{\text{drug quantity}}\times 100\% \end{array}$$ (1)

$$\begin{array}{c} \text{Drug loading rate}\;\left(\%\right)=\\ \frac{\text{sample quantity}\;-\;\text{free drug quantity}}{\text{sample quantity}+\text{material quantity}}\times 100\% \end{array}$$ (2)

The release rates of FS@MMS-GEM in phosphate-buffered saline (PBS) solutions at different pH values were investigated using the dialysis method. The nanomedicine agent was first dispersed in deionized water to prepare a 1 mg/ml solution, after which 1-ml solutions of GEM, fasudil, and FS@MMS-GEM were precisely measured and placed in dialysis bags. The dialysis bags were then placed in separate containers containing 20 ml of PBS release medium at pH 7.4, 6.8, and 5.5, and the in vitro release was investigated using constant-temperature oscillation (100 r/min) at 37 °C. Samples were taken at different time points (0, 1, 2, 4, 8, 12, 24, and 48 h), and the same volume of fresh media was added. The samples were then analyzed by UV–Vis spectrometry at 490 nm, and the percentages of total GEM and fasudil released were calculated according to the standard curve.

### Intracellular and extracellular MRI of CAFs and Panc02 cells after nanomedicine treatment

The ability of the nanotension relief agent to be detected via magnetic resonance imaging (MRI) utilizing a 3.0-T MRI scanner was assessed. The nanotension relief agent was diluted to various concentrations (1.03, 0.51, 0.13, 0.06, 0.03, and 0.002 mM) with PBS. *T*_2_-weighted images and *T*_2_ relaxation times of the nanomedicine were acquired using a *T*_2_ spin-echo sequence. The parameters were set as follows: matrix = 384 × 256, repetition time (TR) = 2,320 ms, and echo time (TE) = 10, 20, 40, 60, 80, 100, and 120 ms. *T*_2_ values corresponding to each concentration were measured using the *T*_2_ mapping function of the workstation. The curves of 1/*T*_2_ and the iron concentration were fitted linearly, and the slope was the relaxation rate of the nanotension relief agent. The ability of the intracellular nanomedicine to be detected via MRI was assessed in CAFs and Panc02 cells. CAFs were generated from NIH-3T3 cells induced with TGF-β (10 ng/ml) for 96 h. Then, the CAFs were inoculated in 6-well plates (5 × 10^5^ per well). Once the materials were attached, the nanotension relief agent (100 μg/ml) was added to the wells, and the PBS-treated wells were used as the control group. The remaining cells in the wells were washed with PBS and collected after incubation for 0.5, 1, 2, and 4 h. The cells were placed in 2-ml Eppendorf tubes, and *T*_2_-weighted images were acquired using the *T*_2_ fast spin echo sequence. The parameters used were as follows: matrix = 384 × 256, TR = 2,380 ms, TE = 72.1 ms, field of view = 16, and number of excitations = 4. The signal changes were calculated using the following equation: Signal changes = SIc − SIi, where SIi and SIc are the signal intensities of CAFs untreated or incubated, respectively, for 0.5, 1, 2, or 4 h.

### FS@MMS uptake in vitro

Prussian blue staining was used to assess cell uptake ability. CAFs were cultured in 6-well plates (5 × 10^5^ per well) and incubated with FS@MMS (100 μg/ml) for 0.5, 1, 2, or 4 h. The group treated with PBS served as the control group. The cells were subsequently collected and stained with Prussian blue.

### FS@MMS reduces CAF contractile tension in vitro

To assess the impact of nanomedicine on tension-related proteins in CAFs, CAFs were seeded into 6-well plates (5 × 10^5^ cells per well) and treated with fasudil (12.5 μg/ml), MMS (100 μg/ml), or the nanotension relief agent (100 μg/ml) for 24 h. Protein extraction was performed using cell lysate supplemented with 1% phenylmethanesulfonyl fluoride and 1% calcineurin inhibitor. Western blotting was conducted following standard protocols. The polyvinylidene fluoride membranes were incubated with anti-phosphorylated myosin light chain (anti-p-MLC) and anti-phospho-myosin phosphatase (anti-p-MYPT) rabbit monoclonal antibodies (1:1,000) overnight and then incubated with a rabbit immunoglobulin G (H+L) secondary antibody (1:10,000) for 2 h to visualize the protein bands. Glyceraldehyde-3-phosphate dehydrogenase expression served as an internal standard for normalizing the expression of other proteins. The signals were detected using enhanced chemiluminescence. Protein expression levels were subsequently quantified using the ImageJ software.

To investigate the impact of the nanotension relief agent on F-actin, CAFs were plated in 24-well plates (1 × 10^5^ cells per well) and incubated with various drug combinations for 24 h. F-actin was stained with fluorescein isothiocyanate (FITC)-labeled phalloidin (1:500) after the nuclei were stained with 4′,6-diamidino-2-phenylindole (DAPI), and the morphological alterations of F-actin were observed and documented under a microscope.

To investigate the effects of nanomedicine on the expression of stress fibers in CAFs, CAFs were seeded into 24-well plates at a density of 1 × 10^5^ cells per well and subsequently incubated for 24 h with diverse combinations of drugs. After cell fixation and permeabilization, the cells were blocked with goat serum, incubated with an anti-α-SMA rabbit monoclonal antibody (1:500) overnight and labeled with Alexa Fluor Plus 594 (1:500). The nuclei were restained with DAPI, and the expression of intracellular stress fibers was observed and recorded using a microscope.

### Cytotoxicity of FS@MMS-GEM in vitro

#### CCK-8 assay

The toxicity of the nanomedicine on Panc02 cells was assessed using a CCK-8 assay. Panc02 cells were cultured in 96-well plates (1 × 10^4^ cells per well) for 24 h. The cells were then treated with fresh culture media containing various concentrations (100, 25, or 6.25 μg/ml) of the nanotension relief agent, GEM, GEM-MMS, or FS@MMS-GEM with different concentrations (25, 6.25, or 1.56 μg/ml) of GEM for another 24 h. CCK-8 solution was added to each 96-well plate (100 μl per well) and incubated with cells at 37 °C for 40 min. The optical density at 450 nm (OD_450_) was measured, and cell viability was calculated using the following equation:

$$\text{Cell viability}=\frac{\text{experimental}\;\mathrm{OD}\;-\;\text{blank}\;\mathrm{OD}}{\text{control}\;\mathrm{OD}\;-\;\text{blank}\;\mathrm{OD}}\times 100\%$$ (3)

where OD refers to the optical density; the blank group contained no cells, and the control group included cells treated without drugs.

#### Live/dead staining

Live/dead staining was employed to evaluate the cytotoxicity of FS@MMS-GEM on Panc02 cells. Panc02 cells were seeded into 96-well plates at a density of 1 × 10^5^ cells per well and subsequently incubated with diverse combinations of drugs after 24 h. Subsequently, calcein-AM/propidium iodide (PI) (1.5 μM/2 μM) was added to the treated cells for 25 min. Finally, the cells were examined under a fluorescence microscope. For apoptosis analysis, Panc02 cells were cultured in 6-well plates and incubated with different combinations of drugs for 24 h. Annexin V–FITC/PI staining was performed, and the quantification of cell apoptosis was assessed via flow cytometry.

#### TEM observation

To investigate the mechanism of Panc02 cell death, the nuclear morphology of the cells was further observed via electron microscopy. After being treated with different combinations of drugs for 24 h, Panc02 cells were collected and observed via TEM.

### Drug penetration in 3-dimensional Panc02/NIH-3T3 multicellular tumor spheroids

The procedure for establishing Panc02/NIH-3T3 multicellular tumor spheroids (MTSs) was as follows: Panc02 and NIH-3T3 cells were suspended in complete medium supplemented with 0.24% methylcellulose at a ratio of 2:1. The cellular concentration was adjusted to reach a density of 8 × 10^5^ cells/ml. The suspension was subsequently added to the lid of a 96-well plate, with 25 μl per drop. After 48 h of suspension culture, the cell spheres were transferred to a 96-well plate containing 1% agarose gel at the bottom for an additional 4 d, during which time Panc02/NIH-3T3 MTSs formed.

To explore the ability of different drugs to penetrate the MTSs, the cell spheres were coincubated with PBS, GEM-MMS (100 μg/ml), the nanotension relief agent (100 μg/ml), or FS@MMS-GEM (100 μg/ml) for 24 h, after which the MTSs were washed with PBS 3 times. The MTSs from each group were incubated with FITC–dextran (molecular weight 4,000) for 40 min. The area of green fluorescence within the MTSs was observed using an upright fluorescence microscope. The MTSs were embedded in 3.5% agarose and subsequently frozen-sectioned. The nanomedicine present in the sections was stained with Prussian blue, and the distribution of blue-stained areas within the tissue was observed.

To further elucidate the effects of drugs on the MTSs, frozen sections of cell spheres were stained with α-SMA and collagen I for immunofluorescence analysis. The expression levels of α-SMA and collagen I in each group were observed using fluorescence microscopy and imaged accordingly.

### FS@MMS-GEM relieves TSS and enhances drug penetration in PDAC tissue

To assess the effectiveness of FS@MMS-GEM in reducing solid stress within tumor tissue, Panc02/NIH-3T3 tumor-bearing mice (5 mice/group) were treated with normal saline (NS), free GEM (2.5 mg/kg), free GEM (2.5 mg/kg) + free fasudil (1.25 mg/kg), the nanotension relief agent (10 mg/kg), GEM-MMS (10 mg/kg), or FS@MMS-GEM (10 mg/kg) once every 3 d. After 15 d of treatment, the tumor tissue of each group was dissected and cut along the long axis of the tumor tissue, with the cut depth being 80% of the short axis. The tissue was then immersed in Hanks’ buffer for 10 min. The distance between these 2 cut edges and the incision depth were measured, and the ratio of the 2 measured values was used to evaluate the TSS.

To evaluate the ability of the nanomedicine to penetrate tumor tissue, we monitored changes in tumor tissue in Panc02/NIH-3T3 tumor-bearing mice during the initial and final drug injections. In this study, tumor-bearing mice in the nanotension relief agent (10 mg/kg) and FS@MMS-GEM (10 mg/kg) groups underwent MRI before and 2 h after treatment. The scanning parameters were as follows: matrix size = 256 × 256, TR = 1,720 ms, TE = 85 ms, field of view = 10 cm, and number of excitations = 4.

To assess the accuracy of MRI for evaluating drug penetration, the tumor tissue and vital organs of mice were collected in the 2 treatment groups after the initial MRI session. The iron content in these samples was determined using inductively coupled plasma–mass spectrometry (ICP–MS) to analyze the biological distribution and accumulation of the nanomedicine. The iron content was expressed as a percentage of the injection dose (%ID) or was normalized to the tissue weight (%ID/g). In addition, 24 h after the last injection, the tumor tissues of the mice treated with the nanotension relief agent and FS@MMS-GEM were collected, fixed, embedded in paraffin, sectioned, and stained with Prussian blue to visualize the accumulation of nanomedicine in the tumor tissue.

### FS@MMS-GEM-mediated remodeling of the PDAC stroma

To investigate the mechanism of TSS relief, the dissected tumor tissue was fixed overnight with 4% paraformaldehyde at 4 °C. The tissue was subsequently sliced into 4-μm sections for staining. The tumor sections were subjected to immunofluorescence staining for α-SMA (1:500), collagen I (1:500), and CD31 (1:500), as well as immunohistochemical staining for hypoxia-inducible factor-1α (HIF-1α; 1:200) and Ki67 (1:8,000). The area covered with collagen fibers was visualized via Masson staining.

### In vivo antitumor effects and biosafety

The antitumor efficacy of the nanomedicine was evaluated in a subcutaneous tumor model. Once the tumor volume attained a size of 50 mm^3^, the mice were subsequently allocated at random into 6 distinct groups (*n* = 5 each). The mice in the 6 groups were injected with NS, free GEM (2.5 mg/kg), free GEM (2.5 mg/kg) + free fasudil (1.25 mg/kg), the nanotension relief agent (10 mg/kg), MMS-GEM (10 mg/kg), or FS@MMS-GEM (10 mg/kg) through the tail vein. The tumor volume was measured before every treatment. After the 6 treatments, the tumors of all mice were dissected. The tumor tissue was fixed overnight with 4% paraformaldehyde and cut into 4-μm sections for hematoxylin and eosin (H&E) staining.

The hemolytic activity of the nanomedicine was evaluated through hemolysis experiments. Fresh anticoagulant-treated blood was separated and diluted in PBS (pH 7.4) to isolate red blood cells (RBCs) via centrifugation (1,200 r/min, 10 min). After washing the RBCs with NS, they were diluted to a 2% (v/v) suspension. This suspension was then incubated with FS@MMS-GEM at various concentrations (25, 50, 100, and 200 μg/ml) for 1 h at 37 °C. Following incubation, supernatants were collected and measured at 570 nm.

To evaluate the short-term safety of the treatments in tumor-bearing mice, blood samples were collected from the orbital sinus at 0, 7, and 14 d after intravenous injection of FS@MMS-GEM (10 mg/kg). Whole blood was collected for hematological analysis. After 15 d of treatment, the organs of all mice were dissected, fixed, sectioned, and stained with H&E.

### Statistical analysis

SPSS software version 22.0 was used for statistical analysis. All of the results are expressed as mean ± standard deviation (SD). The data were analyzed using either a *t* test or one-way analysis of variance unless otherwise specified. The Kruskal–Wallis test was used to compare the tumor volumes. When bilateral *P* < 0.05, the difference between groups was considered statistically significant.

## Results

### Synthesis and characterization of FS@MMS-GEM

**Fig. 2.**
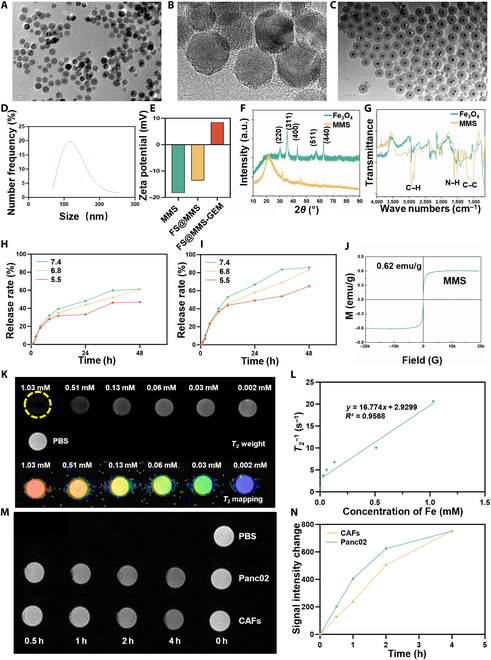
Characterization of FS@MMS-GEM. (A and B) Transmission electron microscopy (TEM) image and high-resolution TEM (HRTEM) image of the Fe_3_O_4_ nanoparticles. (C) TEM image of the MMS nanocarrier. (D) Size of the MMS nanocarrier. (E) Zeta potentials of MMS, FS@MMS, and FS@MMS-GEM in 10 mM Hepes buffer (pH 7.4). (F) X-ray diffraction (XRD) patterns of the Fe_3_O_4_ and MMS nanocarriers. (G) Fourier transform infrared spectroscopy (FTIR) spectra of the Fe_3_O_4_ and MMS nanocarriers. (H and I) Release rates of GEM and fasudil from MMS at various pH values and time intervals. (J) Saturation magnetization of the MMS nanocarrier. (K) *T*_2_-weighted images and *T*_2_ mapping of different concentrations of the nanotension relief agent (FS@MMS) (1.03, 0.51, 0.13, 0.06, 0.03, and 0.002 mM). (L) The fitting curve between 1/*T*_2_ and the concentration of Fe was obtained according to *T*_2_ mapping. (M) Magnetic resonance (MR) images of Panc02 cells and CAFs incubated with the nanotension relief agent (100 μg/ml) for 0, 0.5, 1, 2, and 4 h. (N) As the incubation time between the cells and nanocarrier increased, the signal intensity decreased gradually. PBS, phosphate-buffered saline.

The size and morphology of the synthesized Fe_3_O_4_ and MMS nanocarriers were characterized by TEM and high-resolution TEM, and the results are shown in Fig. 2A to C. The results revealed that the morphology of the synthesized Fe_3_O_4_ nanoparticles was quite uniform and that the particles were approximately 20 nm in size. After the surface of the Fe_3_O_4_ nanoparticles was coated with mesoporous silicon, the thickness of the mesoporous silicon layer was approximately 25 nm. Figure 2D shows that the hydrodynamic diameter of MMS was approximately 105 nm, thus allowing efficient uptake by tumor cells and CAFs via the enhanced permeability and retention effect. The surface charge of MMS was approximately −13.6 mV. However, after loading GEM and fasudil, the surface charge of MMS decreased to approximately 8.45 mV (Fig. 2E).

Furthermore, the structural information was characterized. As shown in Fig. 2F, the pure Fe_3_O_4_ nanoparticles had suitable crystallinity and corresponded with the standard card (PDF no. 19-0629). After coating with mesoporous silicon, the characteristic peak of the Fe_3_O_4_ nanoparticles disappeared, and the broad peak at approximately 21.5° corresponded to the structure of the mesoporous silicon layer. The FTIR spectra of the Fe_3_O_4_ and MMS nanocarriers are shown in Fig. 2G. After coating with mesoporous silicon, the peaks at 2,924.7 and 2,853.8 cm^−1^ corresponded to C–H stretching vibrations; the peaks at 1,603.8 and 1,507.8 cm^−1^ corresponded to N–H bending vibrations; and the peak at 1,041 cm^−1^ corresponded to C–H stretching vibrations. Therefore, the FTIR data indicated functional groups and mesoporous silicon on the surface of the Fe_3_O_4_ nanoparticles.

To verify the drug loading capacity of FS@MMS-GEM, we used UV spectrometry to determine the drug loading rate and encapsulation rate of FS@MMS-GEM based on the standard curves of GEM and fasudil absorbance. The results revealed that the encapsulation rate of GEM was 69.5%, whereas the drug loading rate was 23.2%. For fasudil, the encapsulation rate was 84.5%, and the drug loading rate was 28.2%. As shown in Fig. 2H and I, FS@MMS-GEM exhibited a biphasic release pattern, and GEM and fasudil were released rapidly in the first 4 h, followed by sustained slow release of the drugs. The release curves at all pH values revealed that the drug release rate increased with increasing acidity. Specifically, at a pH of 6.8, the cumulative release rates of GEM and fasudil within 2 h were 6.8% and 5.6%, respectively, and by 48 h, the cumulative release rates reached 83.1% for GEM and 61.1% for fasudil. When the pH was 5.5, the release rates of both drugs were even greater. This drug release pattern not only reduced the damage to normal cells caused by high concentrations of the drugs but also improved the targeting and release of the drugs into PDAC tissue, thereby increasing their effectiveness.

To evaluate the ability of the nanotension relief agent to be detected by MRI, we used a vibrating-sample magnetometer to test the magnetic properties of the Fe_3_O_4_ and MMS nanocarriers, and the results are shown in Fig. S1 and Fig. 2J. The peak magnetization values for Fe_3_O_4_ and MMS were 55.92 and 0.62 emu/g, respectively, indicating superparamagnetism. To assess the ability of the nanotension relief agent to be imaged extracellularly, *T*_2_-weighted MRI and *T*_2_ mapping were conducted at various concentrations ranging from 0.002 to 1.03 mM of the nanotension relief agent. An apparent linear relationship was observed between the transverse relaxivity and the concentration of the nanocarrier (*r*_2_ = 16.77 mM^−1^ s^−1^), indicating that the MRI contrast was dependent on the iron concentration (as shown in Fig. 2K and L). To further evaluate the practical application of the nanocarrier for MRI, mouse embryonic fibroblasts (NIH-3T3) were coincubated with TGF-β for 96 h to obtain CAFs, and *T*_2_-weighted magnetic resonance scanning was performed on Panc02 cells and CAFs that had been coincubated with the nanotension relief agent. As the incubation time increased, the signal intensity of Panc02 cells and CAFs gradually decreased. Moreover, the amplitude of the change in signal intensity in CAFs was lower than that in Panc02 cells within the first 2 h, and the signal strength decreased by 28% and 35%, respectively (Fig. 2M and N). These results suggested that the nanotension relief agent has good iron-concentration-dependent MRI capabilities both inside and outside the cells.

### Nanotension relief agents rapidly transform high-tension CAFs into low-tension CAFs

**Fig. 3.**
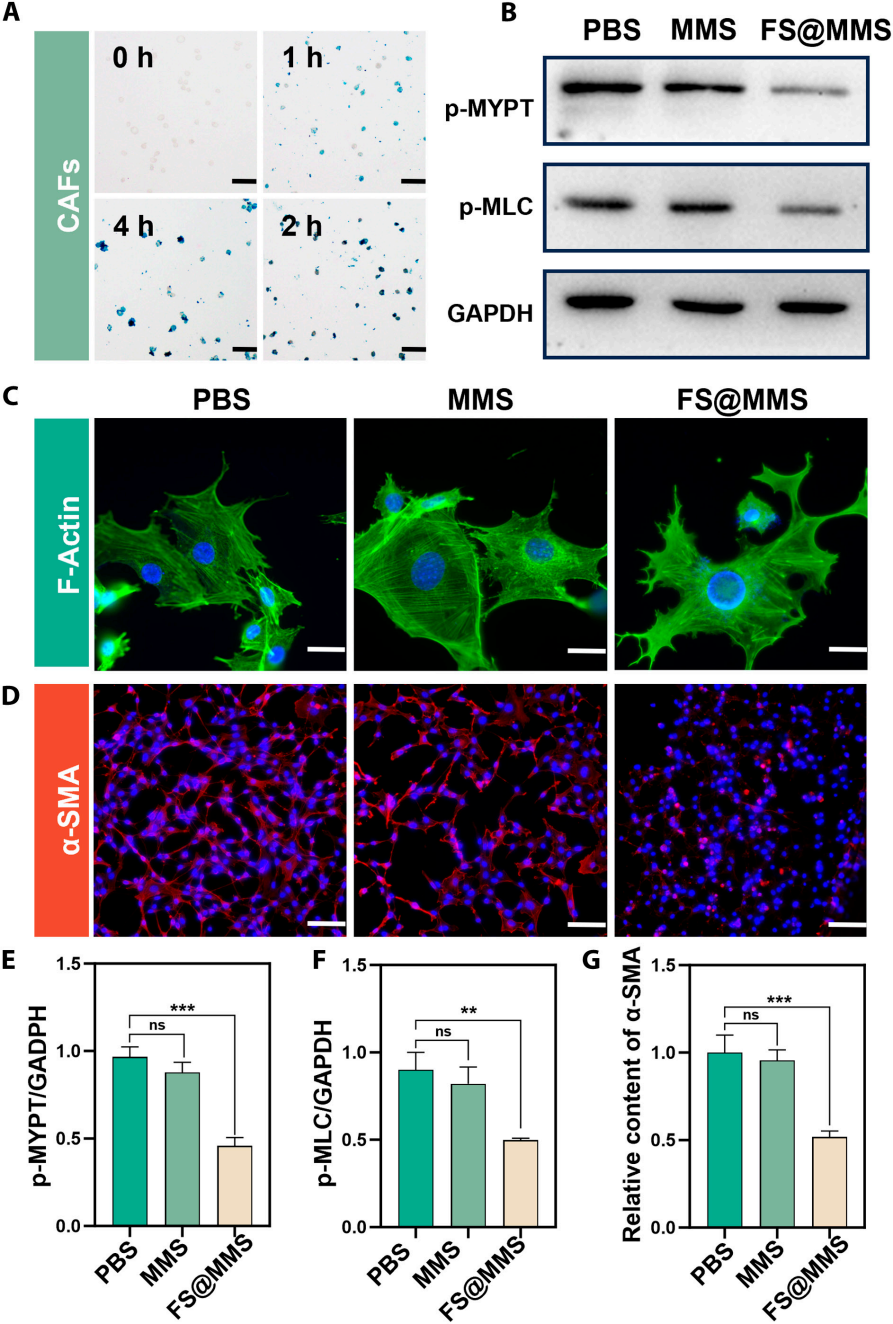
Nanotension relief agents (FS@MMS) rapidly transform high-tension CAFs into low-tension CAFs. (A) Prussian blue staining of CAFs incubated with the nanotension relief agents (100 μg/ml) for 0, 1, 2, and 4 h (scale bars: 100 μm). (B, E, and F) Protein expression and semiquantitative analysis of p-MLC and phospho-myosin phosphatase (p-MYPT) in CAFs from different treatment groups. (C) Morphological manifestations of F-actin in CAFs treated with different drugs (scale bars: 50 μm). (D and G) The expression of α-SMA in the different treatment groups (scale bars: 100 μm). Data are presented as mean ± SD (*n* = 3). ns, not significant; **P < 0.01; ***P < 0.001. GAPDH, glyceraldehyde-3-phosphate dehydrogenase.

For effective therapeutic intervention, efficient internalization of nanomedicine by cells is essential. Prussian blue staining was performed on CAFs incubated with nanotension relief agents for 4 h. As shown in Fig. 3A, the intensity of intracellular blue staining increased gradually with prolonged incubation time, suggesting that CAFs have substantial levels of endocytosis of nanotension relief agents, which is the key to the therapeutic effect of nanotension relief agents.

Activated CAFs, which are in a state of heightened contractile tension, play a crucial role in sustaining elevated TSS within PDAC tissue [6]. The structural cornerstone of CAF contractility lies in the binding of myosin to the actin cytoskeleton. To explore the molecular mechanisms underlying the effects of nanotension relief agents, we examined their effects on the expression levels of 2 key proteins, p-MLC and p-MYPT, both of which can reflect the activity of the ROCK protein and the CAF contractility. As presented in Fig. 3B, E, and F, the analysis revealed distinct alterations in the expression patterns of p-MLC and p-MYPT across various treatment groups. Notably, treatment with the nanotension relief agents reduced the levels of both p-MLC and p-MYPT compared with those in the PBS group. In contrast, the MMS treatment groups, which served as a comparison, did not exhibit statistically significant changes in these protein levels (*P* > 0.05). These findings strongly suggest that the nanotension relief agent can suppress the phosphorylation of MLC by inhibiting ROCK, thereby inhibiting the contractile forces generated by the actomyosin interaction in CAFs [24].

Intracellular F-actin and α-SMA are pivotal proteins in CAFs that facilitate the formation of contractile actin fibers. The stability of these proteins is critically maintained through their binding to p-MLC [25,26]. To assess the influence of modulated p-MLC expression on F-actin dynamics, we used an FITC-labeled cyclopeptide to visualize the F-actin of CAFs incubated with PBS, MMS, or nanotension relief agents for 24 h. Notably, in the groups treated with nanotension relief agents, F-actin exhibited a disorganized appearance, accompanied by shortened and unstable cellular protrusions, in stark contrast to the regular and orderly arrangement observed in the PBS and MMS groups (Fig. 3C). This phenomenon arises from the reduced availability of myosin II for stress fiber formation and maintenance of stress fiber tension, leading to cytoskeletal depolymerization; in response to this alteration in tension, the cells formed an increased number of protrusions to compensate [16]. Furthermore, we explored the expression of α-SMA in CAFs from each treatment group using cellular immunofluorescence staining, with cellular α-SMA marked by red fluorescence. Compared with that in the PBS and MMS groups, the expression of α-SMA in the nanotension relief agent groups was lower (Fig. 3D and G). These results demonstrated that decreased expression of p-MLC can inhibit the stability of F-actin and α-SMA in CAFs. Collectively, our findings suggest that nanotension relief agents, by suppressing MLC phosphorylation, impede the ongoing assembly of actin fibers, thereby converting high-tension CAFs into low-tension CAFs.

### Cytotoxicity of FS@MMS-GEM in vitro

**Fig. 4.**
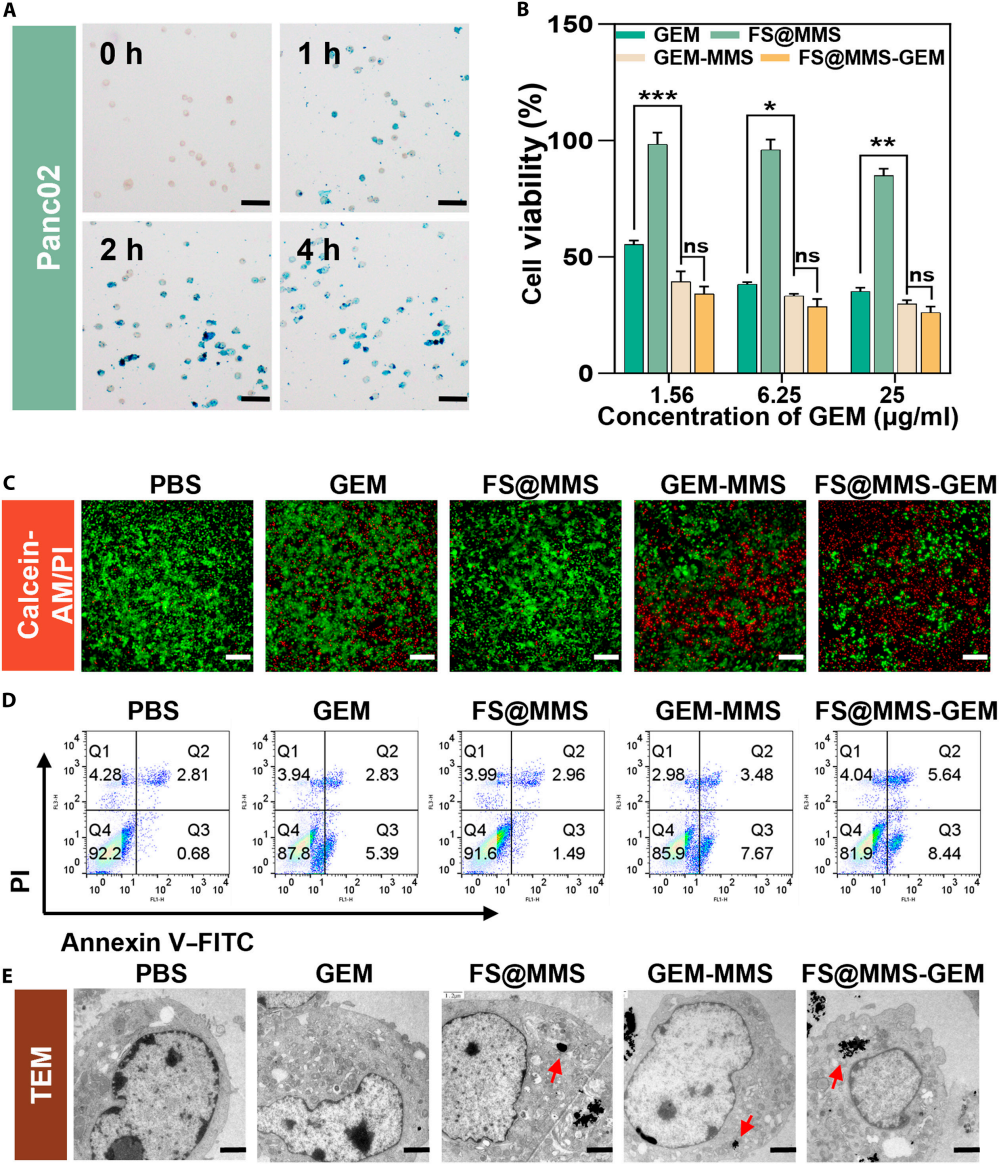
Cytotoxicity of FS@MMS-GEM in vitro. (A) Prussian blue staining of CAFs incubated with FS@MMS-GEM (100 μg/ml) for 0, 1, 2, and 4 h (scale bars: 50 μm). (B) Viability of Panc02 cells incubated with different drug combinations at different concentrations for 24 h. (C) Fluorescence images of Panc02 cells stained with calcein-AM and propidium iodide (PI) after treatment with different formulations (red: dead/late apoptotic cells; green: viable cells; scale bars: 100 μm). (D) Flow cytometric analysis of Panc02 cells after staining with annexin V–fluorescein isothiocyanate (FITC) and PI. (E) Nuclear changes in Panc02 cells induced by different combinations of drugs were observed via TEM; arrows indicate the nanomedicine engulfed by Panc02 cells, and the scale bars represent 1.2 μm. Data are presented as mean ± SD (*n* = 3). ns, not significant; *P < 0.05; **P < 0.01; ***P < 0.001.

To evaluate the cytotoxicity of FS@MMS-GEM on PDAC cells, Prussian blue staining was performed on Panc02 cells incubated with FS@MMS-GEM for 4 h. As shown in Fig. 4A, Panc02 cells exhibited substantial endocytosis of FS@MMS-GEM in a time-dependent manner. The viability of Panc02 cells results showed that the viability of Panc02 cells decreased with increasing GEM, GEM-MMS, and FS@MMS-GEM concentrations, whereas nanotension relief agents alone (100 μg/ml) had no inhibitory effect on the viability of Panc02 cells (Fig. 4B). In addition, compared with GEM alone, GEM-MMS and FS@MMS-GEM at the same GEM concentration had more significant inhibitory effects on Panc02 cells, but there was no significant difference in the tumor cell inhibitory effect between the 2 groups. Therefore, nanotension relief agents alone had no cytotoxic effect on the cells, indicating that GEM played a major toxic role in FS@MMS-GEM.

To verify the effect of FS@MMS-GEM on tumor cells in vitro, calcein AM/PI staining was used to label live and dead cells treated with different combinations of drugs. At the same GEM concentration, the death rates of Panc02 cells in the GEM-MMS and FS@MMS-GEM treatment groups were significantly higher than that in the free GEM group, and the nanotension relief agent alone had almost no cytotoxic effects on PDAC cells (Fig. 4C). The flow cytometry results revealed that the cell death rates in the PBS group and the group treated with the nanotension relief agent alone were 3.49% and 4.45%, respectively, which were lower than those in the GEM, GEM-MMS, and FS@MMS-GEM groups (8.22%, 11.15%, and 14.08%, respectively) (Fig. 4D). Therefore, GEM was the key component in the killing of Panc02 cells in this study, and the nanotension relief agent (100 μg/ml) had almost no cytotoxicity. To verify the therapeutic mechanism of FS@MMS-GEM in vitro, we scanned tumor cells in different treatment groups via electron microscopy. The results revealed that GEM caused only early apoptotic changes, such as nuclear membrane shrinkage, whereas the GEM-MMS and FS@MMS-GEM groups presented marked apoptotic bodies and chromatin marginalization at the same dose, which are typical manifestations of cell apoptosis. Notably, many nanoparticles could be detected in Panc02 cells via electron microscopy (Fig. 4E). These findings suggested that FS@MMS-GEM significantly induced apoptosis and promoted PDAC cell death.

### Amplified TSS relief of FS@MMS-GEM in Panc02/NIH-3T3 MTSs

**Fig. 5.**
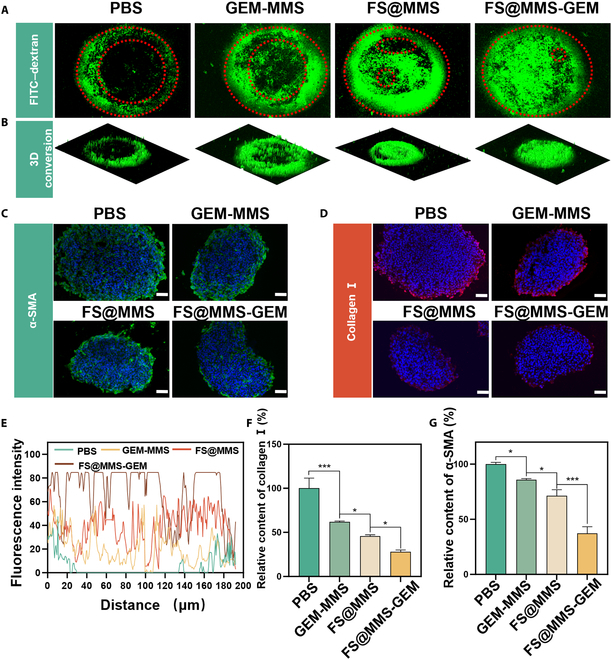
Amplified TSS improvement of FS@MMS-GEM in Panc02/NIH-3T3 multicellular tumor spheroids (MTSs). (A) FITC–dextran penetration and (B) corresponding 3-dimensional (3D) conversion into Panc02/NIH-3T3 MTSs in the different groups. (E) Fluorescence intensity distribution along the diameter of the Panc02/NIH-3T3 MTSs. (C and F) Images of α-SMA expression and differences in α-SMA expression among the different groups. (D and G) Images of collagen I expression and differences in collagen I expression among the different groups (scale bars: 100 μm). The data are presented as mean ± SD (*n* = 3). *P < 0.05; ***P < 0.001.

To further explore the efficacy of FS@MMS-GEM in relieving TSS and enhancing drug penetration within PDAC tissue during brief therapeutic interventions, we established Panc02/NIH-3T3 MTSs as a refined model to more precisely mimic drug penetration within tumor tissue [27]. Using FITC–dextran staining, we systematically investigated the effects of various drug formulations on improving MTS penetration efficiency. Figure 5A shows that, compared with the infiltration area in the PBS group, the GEM-MMS group exhibited a confined infiltration zone. Conversely, both the nanotension relief agent groups and the FS@MMS-GEM group manifested an expansion in the infiltration area, with the latter demonstrating a remarkably superior effect, penetrating through the entire spheroid volume. The 3-dimensional conversion in Fig. 5B clearly illustrates the spatial distribution of FITC–dextran within the MTSs. Quantitative fluorescence intensity analysis, presented in Fig. 5E, underscores the superiority of the FS@MMS-GEM group, yielding the most intense fluorescence signal within the central region of the MTSs. To further validate these findings, Prussian blue staining was employed to assess the nanomedicine diffusion depth within the MTSs. Notably, the FS@MMS-GEM treatment group presented the most extensive blue staining area, which aligned seamlessly with the fluorescence staining results (Fig. S2). Collectively, these observations imply that the integration of nanotension relief agents and the FS@MMS-GEM system fosters the creation of relaxed interstitial channels, thereby facilitating rapid and profound drug penetration into the MTSs, even within a short treatment duration [28].

To further explore the underlying mechanism by which FS@MMS-GEM enhances the drug penetration efficiency within MTSs, we conducted immunofluorescence staining on sectioned cell spheroids to examine the expression levels of α-SMA and collagen I in tumor tissue. α-SMA serves as a hallmark for contractile CAFs, providing a surrogate measure of their contractile force in PDAC. Figure 5C and F show that α-SMA expression was concentrated at the periphery of the spheroids. Compared with that in the PBS group, the expression level of α-SMA in the GEM-MMS and nanotension relief agent groups decreased. Remarkably, the FS@MMS-GEM treatment group exhibited an even more pronounced decrease, suggesting a superior capacity to modulate CAF contractility. Moreover, collagen I reflects the tensile resistance of the PDAC interstitium. The immunofluorescence staining results for collagen I were consistent with the observed difference in α-SMA expression (Fig. 5D and G). The expression level of collagen I in the FS@MMS-GEM treatment group was lower than that in the GEM-MMS and nanotension relief agent treatment groups. These findings confirmed that FS@MMS-GEM can prevent the continued secretion and accumulation of extracellular collagen fibers more effectively, thereby fostering the formation of relaxed interstitial tracts.

We observed that, compared with the PBS control, GEM-MMS treatment led to a notable reduction in α-SMA and collagen I expression in Panc02/NIH-3T3 MTSs. This reduction may be attributed to the nonspecific killing of tumor cells in MTSs caused by GEM-MMS, which weakens the mechanical stimulation of CAFs. Consequently, this indirect effect inhibited the continuous synthesis of α-SMA and the secretion of collagen fibers. In contrast, FS@MMS-GEM was found to be more efficacious in modulating CAF tension by down-regulating p-MLC levels. This targeted approach resulted in a more pronounced alleviation of contractile tension and ECM accumulation. The combined effect of tumor-killing capabilities and enhanced TSS-relief characteristics of FS@MMS-GEM synergistically loosened the tumor stroma, thereby facilitating enhanced drug penetration into the tumor microenvironment.

### The antitumor effects and enhanced TSS-relief characteristics of FS@MMS-GEM in PDAC tissue

**Fig. 6.**
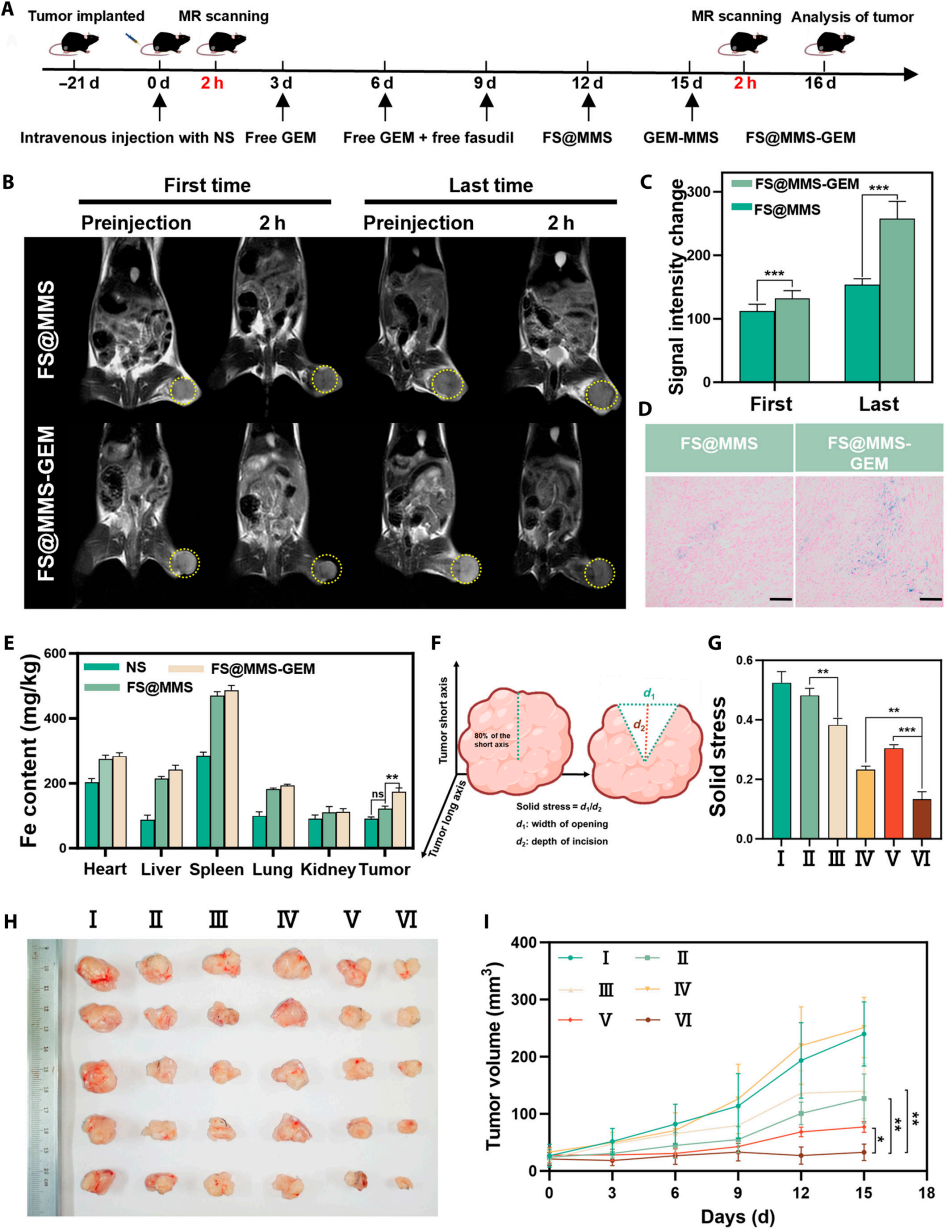
Evaluation of the penetration efficiency and therapeutic effect of FS@MMS-GEM in PDAC tissue. (A) Diagram of the in vivo treatment process. (B) Real-time MR image of the tumor tissue 2 h before and after the first and last tail vein injections of nanotension relief agents (FS@MMS) and FS@MMS-GEM. (C) Statistical analysis of the changes in signal intensity before and after treatment with the nanotension relief agents and FS@MMS-GEM treatment groups at the first and last time points. (D) At 24 h after the last tail vein injection of nanotension relief agents and FS@MMS-GEM, the tumor tissue was stained with Prussian blue (scale bars: 100 μm). (E) The Fe content in major organs and tumor tissues of mice 24 h after the first injection of normal saline, the nanotension relief agent, or FS@MMS-GEM. (F) Schematic diagram of the method used to measure the TSS in tumors. (G) After treatment for 15 d, the TSS of the tumor tissue in different groups was measured. (H) Images of tumors from each group after treatment. (I) Changes in tumor volume (I: PBS; II: free GEM; III: free fasudil + free GEM; IV: FS@MMS; V: GEM-MMS; VI: FS@MMS-GEM). The data are presented as mean ± SD (*n* = 5). *P < 0.05; **P < 0.01; ***P < 0.001. NS, normal saline.

In our prior experimental endeavors, we established that FS@MMS-GEM effectively mitigated the contractile tension of CAFs expeditiously, reduced collagen fiber accumulation, and exhibited potent tumoricidal activity in vitro. Herein, we aimed to validate the enhanced tumor-suppressing effects and TSS-relief capabilities of FS@MMS-GEM in a subcutaneous mouse model. Drawing from previous research, we observed that the Panc02/NIH-3T3 coplanted model is more representative of the clinical characteristics of PDAC than the Panc02 tumor-only transplantation model [29]. We administered NS, free GEM, free GEM + free fasudil, the nanotension relief agent, GEM-MMS, or FS@MMS-GEM to Panc02/NIH-3T3 coplanted model mice via the tail vein once every 3 d for a total of 6 times (Fig. 6A).

To explore the penetration efficiency of the nanomedicine in PDAC tissue during treatment, we leveraged MRI to monitor drug accumulation. Notably, nanotension relief agents exhibited a reliable *T*_2_ imaging capability that was contingent upon the iron concentration, thereby enabling MRI to precisely track both the dosage and penetration depth of the drug within PDAC tissues. Specifically, we selected mice treated with nanotension relief agents and FS@MMS-GEM and conducted MRI scans prior to the initial injection and 2 h postinjection. The results revealed a pronounced reduction in PDAC tissue signal intensity postinjection in both treatment groups, with the FS@MMS-GEM group exhibiting a more marked shift in signal intensity (Fig. 6B and C). To further investigate the in vivo biodistribution of the nanomedicine, we dissected tumor tissues and primary organs from mice after injection with NS, the nanotension relief agent, or FS@MMS-GEM and quantitatively analyzed the iron (Fe) content using ICP–MS. Our analysis revealed that nanotension relief agents and FS@MMS-GEM accumulated significantly more in the liver, spleen, and tumor tissue than in other organs (Fig. 6E). Notably, FS@MMS-GEM demonstrated remarkable accumulation specifically within PDAC tissue, corroborating the MRI findings. These discoveries underscore the efficient penetration of FS@MMS-GEM into PDAC tissue within 2 h. When coupled with the drug release experiments mentioned earlier, it becomes evident that less than 10% of the administered drug is released prior to the extensive infiltration of the nanomedicine into PDAC tissues. This finding implies that the time window for the release and subsequent action of the chemotherapeutic agent within FS@MMS-GEM significantly exceeds the duration required for tissue penetration. Consequently, this extended timeframe enables the medication to fully exert its tumoricidal effects while concurrently mitigating off-target effects and associated organ damage that might arise from prolonged treatment.

To gain further insight, we performed MRI scans both before and after the sixth treatment injection. The results convincingly demonstrated a marked reduction in signal intensity for nanotension relief agents and FS@MMS-GEM within PDAC tissue compared with their initial readings. Notably, the FS@MMS-GEM treatment group exhibited an even more pronounced decrease in signal intensity than did the nanotension relief agent group (Fig. 6B and C). Prussian blue staining of tumor tissue from both groups was used to evaluate drug penetration. Our analysis revealed that, compared with the other groups, the FS@MMS-GEM treatment group presented larger and more widely dispersed blue-stained areas, indicative of deeper penetration and greater distance from blood vessels (Fig. 6D). Collectively, these findings underscore a substantial enhancement in drug penetration efficiency within PDAC tissue in the FS@MMS-GEM group following long-term treatment. Specifically, the synergistic combination of nanotension relief agents and GEM within the FS@MMS-GEM nanomedicine significantly augmented its ability to penetrate the tumor microenvironment.

Solid stress always occurs in solid tumors and increases as tumor cells proliferate, CAF tension increases, and ECM protein production increases [30]. To quantify this phenomenon, we employed a well-established methodology to assess the TSS (Fig. 6F) [31]. Our findings revealed that, in comparison with the control group, free GEM administration led to only marginal relief in TSS. However, when GEM was combined with the nanotension relief agent, a marked and statistically significant decrease in the TSS within PDAC tissues was observed. Notably, this effect surpassed that achieved by the nanotension relief agent alone or GEM-MMS, underscoring the enhanced TSS-relief properties imparted by the synergistic combination of the nanotension relief agent and GEM in the FS@MMS-GEM formulation (Fig. 6G). Additionally, the incorporation of nanotension relief agents addressed the pharmacokinetic problem that fasudil had difficulty accumulating in PDAC tissues. Intriguingly, even the GEM-MMS treatment resulted in a modest reduction in TSS, potentially attributable to the nonspecific tumoricidal effects observed over the course of prolonged treatment.

**Fig. 7.**
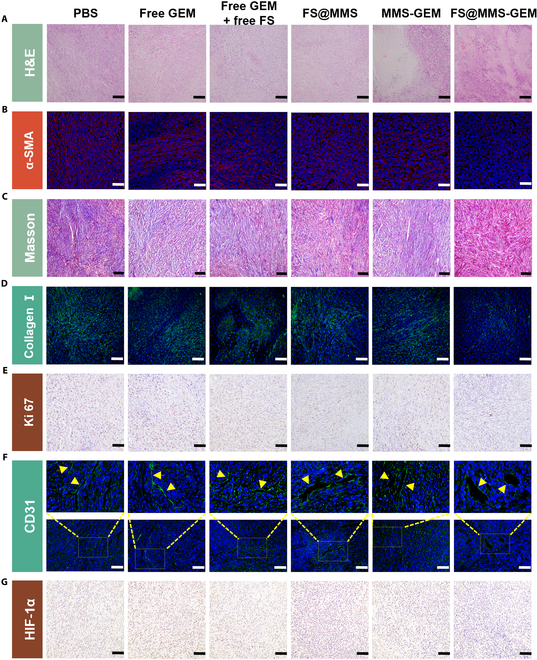
In vivo antitumor effect. (A) Hematoxylin and eosin (H&E) staining of PDAC tissue after treatment (scale bars are 200 μm). (B, D, and F) α-SMA, collagen I and CD31 immunofluorescence staining of PDAC tissue after treatment. (C) Masson staining was performed on PDAC tissue after treatment (scale bars: 100 μm). (E and G) Ki67 and hypoxia-inducible factor-1α (HIF-1α) immunohistochemical staining of PDAC tissue after treatment (scale bars: 100 μm). The data are presented as mean ± SD (*n* = 5).

Based on the potential of FS@MMS-GEM to relieve TSS and promote drug penetration in vivo, we conducted further research to investigate the inhibitory effect of intravenous administration on tumor growth. The tumor size was measured prior to each treatment, as illustrated in Fig. 6H and I. Specifically, the tumor volume in the NS injection group exhibited rapid and unchecked expansion. In comparison, the nanotension relief agent treatment group displayed almost no retardation of tumor growth. Moreover, both the free GEM and free GEM + free fasudil groups managed to decelerate tumor growth to a certain extent, but they failed to arrest its progression. Compared with their free drug counterparts, the MMS-encapsulated drug formulations demonstrated significantly enhanced tumor growth inhibition capabilities. Notably, the FS@MMS-GEM treatment group exhibited a reversal in tumor trajectory, with the tumor tissue volume beginning to decrease on the ninth day of treatment, whereas the GEM-MMS group exhibited a persistent increase in tumor size. These observations collectively underscore the remarkable ability of FS@MMS-GEM to increase drug penetration into PDAC tissues, thereby enhancing therapeutic outcomes. Additionally, an assessment of mouse body weights across all treatment arms revealed no substantial decreases (Fig. S3). Further microscopic analysis through H&E staining of tumor sections corroborated these findings, revealing that the FS@MMS-GEM group presented the most pronounced degree of tissue necrosis, a testament to its heightened antitumor potency (Fig. 7A). Therefore, our nanomedicine, with its enhanced TSS-relief characteristics, could increase the efficacy of antitumor therapy throughout the treatment process.

The TSS within PDAC tissue is substantially influenced by the synergistic action of immortalized tumor cells and a dense stromal microenvironment, as highlighted in previous studies [4]. We further investigated the in vivo mechanism of the increased TSS-relief characteristics exhibited by FS@MMS-GEM. Our findings revealed intriguing patterns: both the free GEM and free GEM + free fasudil groups marginally reduced α-SMA expression, and the FS@MMS-GEM treatment group exhibited notably lower α-SMA expression than did the nanotension relief agent monotherapy and GEM-MMS groups (Fig. 7B and Fig. S4). This observation underscores the pivotal role of the nanotension relief agent in mitigating CAF-mediated tension, which was further enhanced in the FS@MMS-GEM formulation. Intriguingly, GEM-MMS contributed to a decrease in α-SMA expression, albeit to a lesser extent. This may be attributed to the nonspecific cytotoxic effects of GEM-MMS over extended treatment periods, which indirectly led to a depletion of stress fibers in CAFs. However, the individual impact of GEM-MMS pales in comparison to the synergistic, augmented effects observed with FS@MMS-GEM. We further analyzed the fiber content in the interstitium using Masson staining and found that the percentage of interstitial collagen fibers in the FS@MMS-GEM group was the lowest, which was consistent with the finding that α-SMA was expressed in CAFs (Fig. 7C and Fig. S5). Notably, collagen I, the predominant collagen fiber type in the PDAC stroma, plays a pivotal role in preserving tissue stiffness and TSS owing to its remarkable tensile strength [32]. To visualize the collagen I distribution, we employed green fluorescence labeling within PDAC tissues, revealing that the accumulation of collagen I was lower in tumor tissues with low α-SMA expression (Fig. 7D and Fig. S6). To elucidate the impact of our nanomedicine on tumor cell proliferation, we performed Ki67 staining on PDAC tissues. Our findings indicated that treatment with the nanotension relief agent alone had minimal influence on tumor cell proliferation. In contrast, GEM-MMS exhibited a modest inhibitory effect, albeit less pronounced than that achieved with FS@MMS-GEM treatment (Fig. 7E and Fig. S7). Our results convincingly demonstrate that FS@MMS-GEM is highly effective in swiftly mitigating CAF-mediated tension and disrupting the positive feedback loop that reinforces the TSS in vivo. Notably, the sustained penetration of GEM facilitated by long-term therapy amplifies the therapeutic efficacy of FS@MMS-GEM via a positive feedback mechanism, enhancing drug penetration and thus potentiating its anticancer action.

To gain a more intuitive understanding of the impact of FS@MMS-GEM on tumor TSS in PDAC tissue, we assessed blood vessel compression in the tumor tissue. Employing an anti-CD31 antibody for vessel staining, we identified lumen-like structures harboring nuclei as definitive markers of tissue blood vessels. Analogous to the alterations observed in the TSS of tumor tissue across various treatment groups, we discerned a modest augmentation in the short diameter of vessel lumens in the group administered free GEM + free fasudil. Notably, the FS@MMS-GEM treatment cohort presented a more pronounced increase in the short diameter of vessel lumens than did the groups treated with the nanotension relief agent alone and the GEM-MMS group, as illustrated in Fig. 7F and Fig. S8. Furthermore, to assess the hypoxic state within the tissue, we performed immunostaining for HIF-1α. Our findings revealed that tissues characterized by larger vessel lumen diameters more substantially alleviated hypoxia, as depicted in Fig. 7G and Fig. S9. These results collectively suggest that prolonged FS@MMS-GEM treatment can effectively mitigate TSS in PDAC tissue, concomitantly inducing vasodilation. This vasodilation, in turn, facilitates enhanced drug accumulation and oxygen perfusion within the tumor microenvironment, ultimately potentiating its antitumor efficacy.

### Biosafety evaluation

**Fig. 8.**
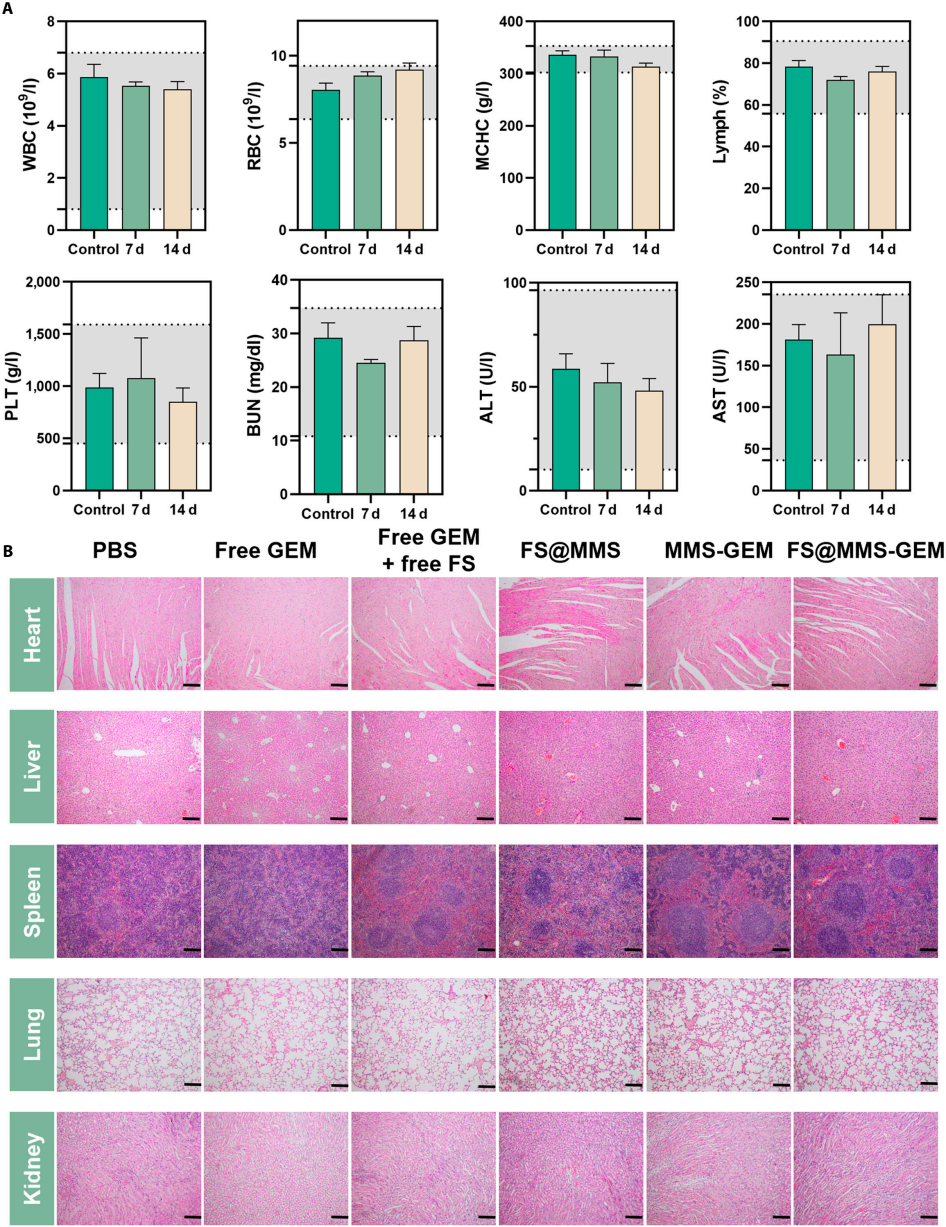
Biosafety evaluation. (A) The results of routine blood, liver, and kidney function biochemical analyses after treatment were normal. (B) H&E staining of the main organs from each group after treatment (scale bars: 200 μm). The data are presented as mean ± SD (*n* = 3). WBC, white blood cells; RBC, red blood cells; MCHC; mean corpuscular hemoglobin concentration; PLT, platelet; BUN, blood urea nitrogen; ALT, alanine transaminase; AST, aspartate transaminase.

To assess the biological safety of the nanomedicine, we investigated the hemolytic toxicity of FS@MMS-GEM. Even when the concentration of FS@MMS-GEM reached 200 μg/ml, the hemolysis rate of RBCs was still less than 5% compared with that of the PBS-treated group (negative control group), indicating that FS@MMS-GEM possessed suitable blood safety (Fig. S10). Hematological analysis and serum biochemical tests were conducted on treated mice 0, 7, and 14 d after the injection of FS@MMS-GEM via the tail vein, and venous blood was collected from the treated mice for hematological analysis and serum biochemical analysis. The results revealed that the nanomedicine had no effect on liver or kidney function or blood cells (Fig. 8A and Fig. S11). Furthermore, the biocompatibility of the nanomedicine was evaluated by examining the main organ of the treated mice via H&E staining. Figure 8B shows that no significant injury or inflammatory damage was observed in the main organs or tissues of the mice in each group, indicating the negligible toxicity of our nanomedicine in vivo. These findings suggest that FS@MMS-GEM has suitable biocompatibility.

## Discussion

The role of CAFs within the stroma of PDAC holds paramount significance in sustaining elevated TSS. High-tension CAFs facilitate remodeling and stiffening of the stroma, which in turn maintains the high-tension phenotype of CAFs, leading to a continuous escalation of TSS within PDAC tissues [33]. While preclinical studies have demonstrated promising results with therapeutic strategies aimed at depleting CAFs to alleviate TSS and reshape the mechanical microenvironment of PDAC, the impact of CAFs on PDAC progression is multiple, as their mere depletion may inadvertently increase cancer invasiveness and metastasis [34,35]. Research by Calvo et al. [16] revealed the emergence of the ROCK protein as a pivotal regulator of the actomyosin cytoskeleton, and transient inhibition of ROCK can disrupt the feedback loop, thereby reversing the high-tension phenotype of CAFs. This discovery presents a therapeutic strategy for modifying the TSS without compromising the stroma. However, the currently employed ROCK inhibitor fasudil has limited stability in blood circulation and struggles to accumulate within tumor tissue, thus posing challenges for its clinical application [36]. In response to these limitations, this study introduces an innovative CAF nanotension relief agent (FS@MMS). This agent is designed to convert high-tension CAFs to low-tension CAFs through modulation of the Rho/ROCK pathway, effectively reducing the TSS of PDAC tissue and significantly augmenting the antitumor effect. This study offers a novel therapeutic approach for targeting CAFs in PDAC.

In this study, we employed mesoporous silicon to encapsulate fasudil via dimercaptosuccinic acid, facilitating its release within the acidic microenvironment of tumor tissue. In vitro experiments revealed that the nanotension relief agent markedly suppressed the expression of p-MLC in CAFs and disrupted actin stability, subsequently mitigating actomyosin-induced cellular contraction in CAFs and reducing tensile force. CAFs rely on mechanosensors, including integrins, focal adhesions, and caveolin-1, to perceive mechanical signals within the tumor tissue [37,38]. The mechanical force exerted by the uncontrolled proliferation of tumor cells and the stiff stroma on CAFs is transduced to the intracellular cytoskeleton. This not only triggers CAF contraction and augments the traction force on the extracellular stroma but also stimulates stroma secretion and remodeling, leading to stroma stiffening and an elevation in the TSS [5,39]. To further investigate the impact of the nanotension relief agent on interstitial tissues, we established Panc02/NIH-3T3 MTSs. Our findings revealed that the nanotension relief agent significantly enhanced the drug penetration efficiency. Moreover, the incorporation of the chemotherapeutic agent GEM synergistically inhibited tumor cell proliferation, resulting in further tissue loosening. This combination therapy broke the vicious cycle of escalating TSS, prevented the overexpression of intrinsic fibers in CAFs, and halted the persistent remodeling and stiffening of the ECM. Notably, this approach diverges from simplistic stroma depletion strategies by enhancing drug penetration without changing the interstitial composition [40,41].

Leveraging the exceptional capacity of FS@MMS-GEM to decrease TSS and eliminate tumor cells, we developed a Panc02/NIH-3T3 coplanted mouse model that mimics the mechanical attributes of PDAC tissue. Under MRI guidance, both the FS@MMS and FS@MMS-GEM formulations demonstrated profound penetration into tumor tissues within 2 h. Notably, at this time, the drug release accounted for less than 10% of the total drug loaded, ensuring that an ample quantity of the therapeutic agent reached deep within the tumor mass. Consequently, our therapeutic approach efficiently reduced the TSS in PDAC tissue and augmented drug penetration efficacy within a brief timeframe. Although numerous nanostrategies have been devised to reverse the CAF phenotype to enhance tissue drug penetration, most are constrained by their effects manifesting only after prolonged treatment periods [42,43]. Transient inhibition of the ROCK protein, however, can induce a phenotypic shift in CAFs and alleviate TSS. Moreover, tumor cells themselves are the primary instigators of TSS within PDAC tissue [4,44]. Merely modulating CAF tension fails to fundamentally alleviate the heightened solid stress state in tumor tissue. To address this, we encapsulated GEM within the nanotension relief agent. During extended treatment, the drug not only penetrated deeply into the tumor tissue but also exhibited potent antitumor activity, leading to more relaxed tumor tissue, reduced intravascular pressure, and a substantial increase in drug perfusion. This series of events fostered a virtuous cycle of TSS reduction, ultimately maximizing antitumor efficacy.

In summary, we have developed a novel nanomedicine, FS@MMS-GEM, aimed at alleviating TSS within PDAC tissues and consequently augmenting the potency of anticancer therapeutic interventions. Our innovative strategy revolves around the inhibition of the Rho/ROCK signaling cascade via the nanotension relief agent (FS@MMS), which effectively inhibits actomyosin contractility in CAFs. This orchestrated transition from high-tension to low-tension CAFs diminishes the mechanical stimuli that CAFs experience, thereby mitigating the excessive accumulation of ECM proteins and decelerating the rapid synthesis of stress fibers in CAFs. These favorable alterations significantly enhance chemotherapeutic drug penetration into the tumor mechanical microenvironment, facilitating deeper penetration and more effective targeting of cancer cells. By integrating its potent tumoricidal properties with its amplified TSS-relief capabilities, FS@MMS-GEM not only directly attacks tumor cells but also optimizes the tumor microenvironment to improve therapeutic outcomes. Furthermore, this effective TSS-relief strategy could be applied to other stroma-rich tumors to address the issue of poor drug penetration in such tumor types.

## Data Availability

The data are available from the corresponding authors on reasonable request.
