# Supplementary material for: Nanotension Relief Agent Enhances Tissue Penetration by Reducing Solid Stress in Pancreatic Ductal Adenocarcinoma via Rho/ROCK Pathway Inhibition
Source: Biomater Res. 2025 Apr 9;29:0173. doi: 10.34133/bmr.0173 (PMC11979343; doi:10.34133/bmr.0173)
Supplement: Supplementary 1 — Figs. S1 to S11 [file bmr.0173.f1.docx]

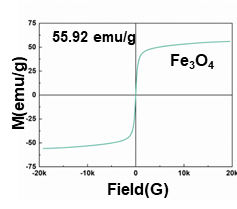


**Fig. S1** The saturation magnetization of Fe_3_O_4_ nanoparticles.


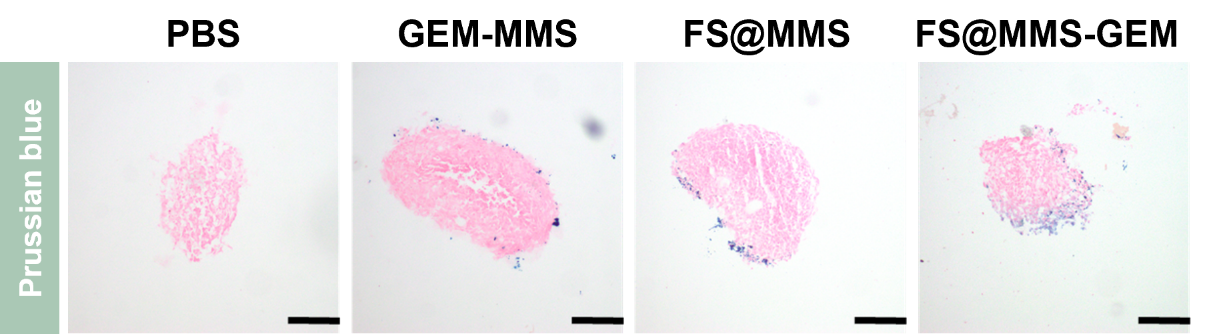


**Fig. S2** The images of Prussian blue staining of different groups. The scale bars are 50 μm


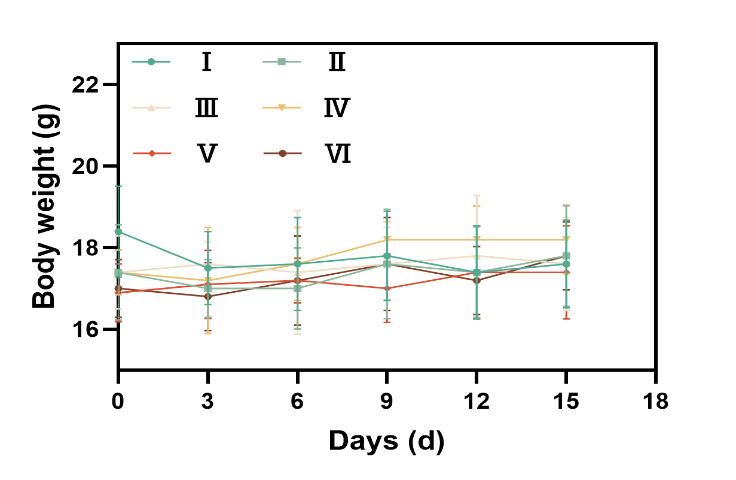


**Fig. S3** Changes in body weight over the course of treatment. (I: PBS, II: free GEM, III: free fasudil+free GEM, IV: nanotension relief agent (FS@MMS), V: GEM-MMS, and VI: FS@MMS-GEM). The data are presented as the mean ± SD (n=5). Significance was assessed by one-way ANOVA followed by the LSD post hoc test.


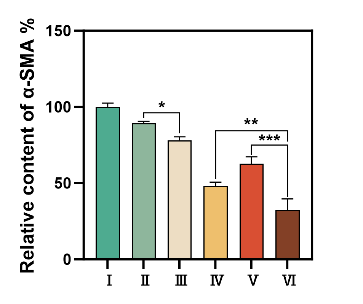


**Fig. S4** Differences in the expression of α-SMA in the different groups. (I: PBS, II: free GEM, III: free fasudil+free GEM, IV: nanotension relief agent (FS@MMS), V: GEM-MMS, and VI: FS@MMS-GEM) The data are presented as the mean ± SD (n=5). Significance was assessed by one-way ANOVA followed by the LSD post hoc test. **P* < 0.05, ***P*< 0.01, ****P* < 0.001


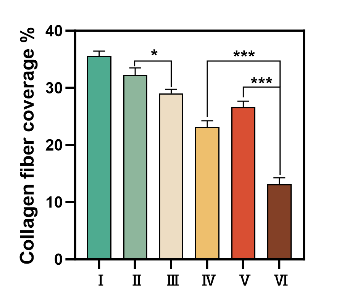


**Fig. S5** Differences in the expression of collagen fiber coverage in the different groups. (I: PBS, II: free GEM, III: free fasudil+free GEM, IV: nanotension relief agent (FS@MMS), V: GEM-MMS, and VI: FS@MMS-GEM) The data are presented as the mean ± SD (n=5). Significance was assessed by one-way ANOVA followed by the LSD post hoc test. **P* < 0.05, ****P* < 0.001


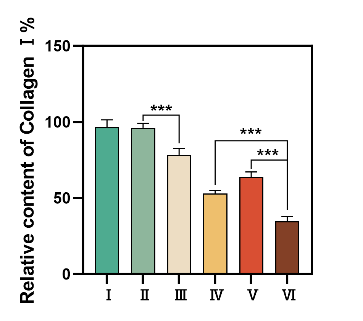


**Fig. S6** Differences in the expression of Collagen Ⅰ in the different groups. (I: PBS, II: free GEM, III: free fasudil+free GEM, IV: nanotension relief agent (FS@MMS), V: GEM-MMS, and VI: FS@MMS-GEM) The data are presented as the mean ± SD (n=5). Significance was assessed by one-way ANOVA followed by the LSD post hoc test. ****P* < 0.001


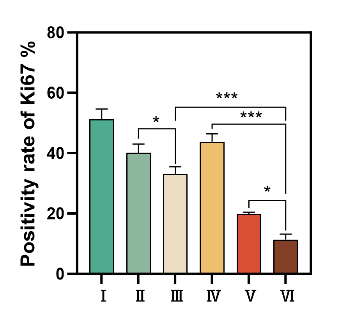


**Fig. S7** Differences in the expression of Ki67 in the different groups. (I: PBS, II: free GEM, III: free fasudil+free GEM, IV: nanotension relief agent (FS@MMS), V: GEM-MMS, and VI: FS@MMS-GEM) The data are presented as the mean ± SD (n=5). Significance was assessed by one-way ANOVA followed by the LSD post hoc test. **P* < 0.05, ****P* < 0.001


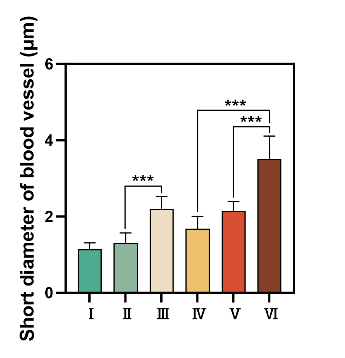


**Fig. S8** Differences of the short diameter of blood vessels in the different groups. (I: PBS, II: free GEM, III: free fasudil+free GEM, IV: nanotension relief agent (FS@MMS), V: GEM-MMS, and VI: FS@MMS-GEM) The data are presented as the mean ± SD (n=5). Significance was assessed by one-way ANOVA followed by the LSD post hoc test. ****P* < 0.001


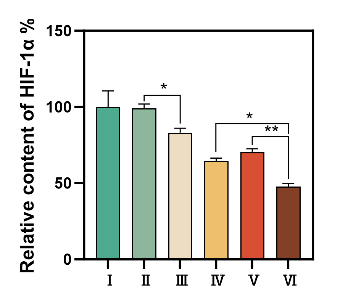


**Fig. S9** Differences of HIF-1α expression in the different groups. (I: PBS, II: free GEM, III: free fasudil+free GEM, IV: nanotension relief agent (FS@MMS), V: GEM-MMS, and VI: FS@MMS-GEM) The data are presented as the mean ± SD (n=5). Significance was assessed by one-way ANOVA followed by the LSD post hoc test. **P* < 0.05, ***P*< 0.01


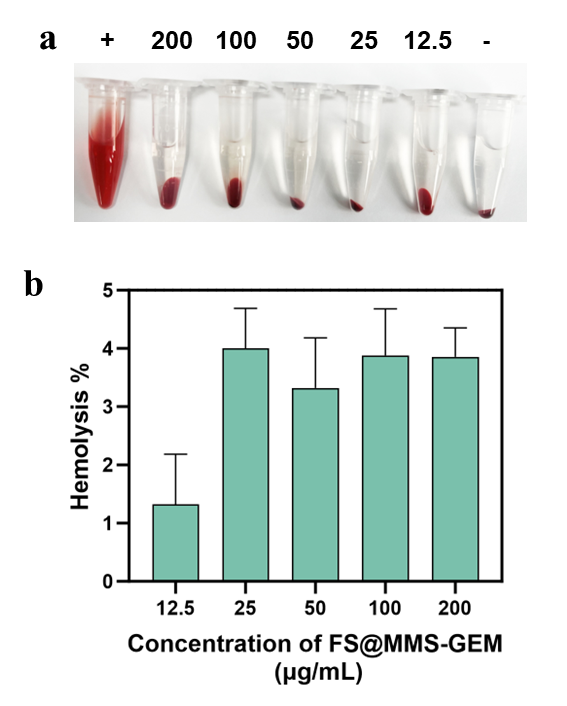


**Fig. S10** (a) and (b) When the concentration of FS@MMS-GEM reached 200 μg/mL, the hemolysis rate of red blood cells remained below 5%. The data are presented as the mean ± SD (n=5).


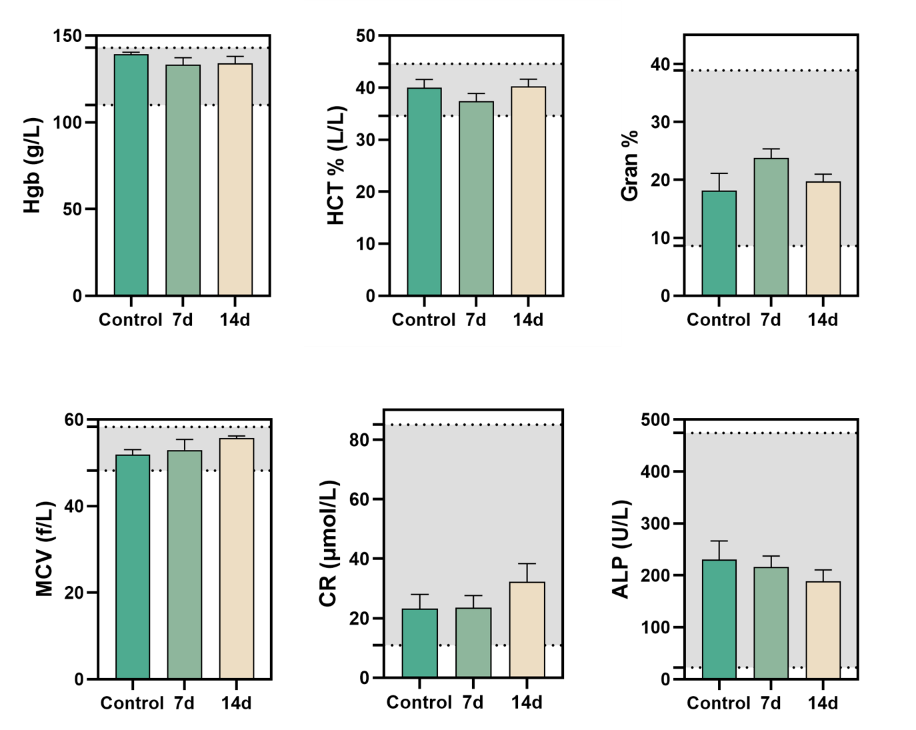


**Fig. S11** After 15 days of treatment with FS@MMS-GEM, the results of routine blood and liver and kidney function biochemical analyses were normal. The data are presented as the mean ± SD (n=3). Hgb, Hemoglobin; HCT%, Hematocrit; Gran%, Granulocyte Percentage; MCV, Mean Corpuscular Volume; CR, Creatinine; ALP, Alkaline Phosphatase.
